# Prolactin Inducible Protein, but Not Prolactin, Is Present in Human Tears, Is Involved in Tear Film Quality, and Influences Evaporative Dry Eye Disease

**DOI:** 10.3389/fmed.2022.892831

**Published:** 2022-06-30

**Authors:** Katharina Jüngert, Friedrich Paulsen, Christina Jacobi, Jutta Horwath-Winter, Fabian Garreis

**Affiliations:** ^1^Department of Functional and Clinical Anatomy, Friedrich-Alexander-Universität Erlangen-Nürnberg (FAU), Erlangen, Germany; ^2^Eyes and Skin Practice Dr. Jacobi, Nürnberg, Germany; ^3^Department of Ophthalmology, Friedrich-Alexander-Universität Erlangen-Nürnberg (FAU), Erlangen, Germany; ^4^Department of Ophthalmology, Medical University of Graz, Graz, Austria

**Keywords:** dry eye disease (DED), prolactin inducible protein, tears, ocular surface, lacrimal gland, lacrimal apparatus, Meibomian gland, prolactin (PRL)

## Abstract

**Purpose:**

Decreased production of the aqueous component of the tear film is an important cause of the development of dry eye disease (DED). Tear production is influenced by hormones and hormone-like factors. Prolactin (PLR), a multifunctional pituitary gland hormone, is regularly present in the lacrimal gland of rats and rabbits. In humans, serum PLR concentration correlates with tear quality. To gain deeper insights of possible effects of PRL, prolactin receptor (PRLR) and prolactin inducible protein (PIP), we analyzed the three proteins in the human lacrimal apparatus and in reflex tears of healthy volunteers as well as patients suffering from DED.

**Methods:**

Gene expression of PRLR and PIP was analyzed by RT-PCR in cadaveric human lacrimal gland and ocular surface tissues, immortalized human corneal epithelial cells (HCE and hTEPI) and human Meibomian gland epithelial cells (HMGECs). At the protein level, the expression and localization of PRL, PRLR and PIP in formalin-fixed paraffin sections of the lacrimal apparatus were studied by immunohistochemistry. In addition, tear fluid from DED patients and healthy volunteers was analyzed by ELISA to determine the concentration of PRL and PIP.

**Results:**

RT-PCR analyses revealed gene expression of PRLR and PIP in human tissue samples of cornea, lacrimal glands, and eyelids, whereas only PIP, but not PRLR, was detectable in immortalized corneal epithelial cells. Immunohistochemistry revealed for the first time the expression and localization of PRL, PRLR, and PIP in human tissues of the lacrimal apparatus and at the ocular surface. PRL and PRLR were detectable in corneal epithelium, lacrimal glands, and Meibomian glands. Reflex tears from DED patients revealed significantly increased PIP concentrations, whereas PRL was undetectable in tears of DED patients and healthy volunteers.

**Conclusion:**

PRL, PRLR, and PIP are found in the lacrimal apparatus and on the ocular surface. PIP, but not PRL, is present in human tears and appears to be involved in the physiology of tear film quality. Our clinical data revealed that PIP may affect tear quality, but further functional analyses are needed to fully elucidate the effects of PRL and PIP-associated factors in tear secretion as well as in the connection of DED.

## Introduction

The ocular surface is covered and protected by the tear film (1). This tear film consists of two layers: an inner muco-aqueous layer built by epithelial cells of the ocular surface, conjunctival goblet cells and acinus cells of the lacrimal gland and accessory lacrimal glands, and an external lipid layer produced by the Meibomian glands in the eye lids (2). Together, the tear film protects the ocular surface, especially the cornea, from drying and supplies it at the same time with oxygen and nutrients. It also allows smooth eyelid movements, protects the ocular surface from mechanical damage and pathogenous microbes, and improves optical properties. Any imbalance in the composition of the tear film components leads to disturbances of the ocular surface and can lead to more or less distinct clinical symptoms of dry eye disease (DED) with limitations of vision (3). According to the TFOS DEWS II report DED is defined as follows: “*Dry eye is a multi-factorial disease of the ocular surface characterized by a loss of homeostasis of the tear film, and accompanied by ocular symptoms, in which tear film instability and hyperosmolarity, ocular surface inflammation and damage, and neurosensory abnormalities play etiological roles*” (4). The two main common causes of DED are: (1) a reduced production of the aqueous component of the tear film, aqueous deficient dry eye (ADDE) and (2) (hyper)evaporative dry eye (EDE) mainly caused by Meibomian gland dysfunction (MGD) (4). Any form of DED can interact with the other form and combinations of ADDE and EDE (mixed dry eye, MDE) occur regularly in patients (5). DED occurs more often in females than in males and is mainly associated with sex hormones (6). Different hormones as well as hormone-like factors have already been shown to have a significant impact on DED. In previous studies, our group showed that the insulin like peptide hormone relaxin 2, insulin like factor and somatostatin occur sex dependent in different concentrations in tears, improve the wound healing capacity of epithelial cells at the ocular surface and modulate immunological processes (7–9).

Prolactin is a multifunctional pituitary gland hormone, that is well-known for its function in mammary gland development and lactation during pregnancy and the breast-feeding period (10). PRL is also built extrapituitary for example in the mammary gland, vascular endothelium, or T-lymphocytes. It has various functions like sodium retention in the small intestine, influence on fear and stress in the brain or immunomodulation of lymphocytes via the JAK-STAT signal transduction pathway that is also used by a variety of cytokines and growth factors (11, 12). PRL is regularly present in tears and is detectable in the lacrimal gland of small laboratory animals like Sprague-Dawley rats and New Zealand White rabbits (6, 13–15). Studies to location and expression of PRL and PRL receptors in human lacrimal apparatus and tears are limited (16, 17). Mathers et al., show a strong negative correlation between serum prolactin level and tear quality in women on hormone replacement therapy (18). Patients suffering from Sjögren's syndrome, a chronic autoimmune disease leading to severe DED, show a higher serum prolactin level compared to healthy volunteers (19). Myal et al. showed that the binding of PRL to the prolactin receptor (PRLR) induces an increased expression of prolactin inducible protein (PIP) (20). Studies have demonstrated, that in different cell types PIP expression is induced by prolactin- and androgen treatment under a not yet fully elucidated mechanism (21, 22). This already suggests that PIP has multiple functions in different physiological, pathophysiological and disease conditions. For example, it is increased in breast and prostate cancer (23) and can therefore be used as a biomarker in breast cancer but also as a prognostic factor for the success of chemotherapy and chance of survival (24). Also, on the ocular surface PIP has a relevant function. As its expression is downregulated in relevant keratoconus cells, tears, saliva and plasma of patients with Keratoconus, it is a novel biomarker for this degenerative disease of the cornea (25–27). Gallo et al. showed that PIP can even be used as a functional biomarker for primary Sjögren's Syndrome in saliva (28). In addition, PIP also plays an important role in immunomodulation and cell-mediate adaptive immunity (21, 23). The present study was undertaken to analyze the expression of PRL, PRLR, and PIP in the human lacrimal apparatus for the first time. In this context, we also measured PRL and PIP concentrations in tears obtained from healthy donors as well as from patients with DED.

## Materials and Methods

### Subjects

All parts of the study were conducted in compliance with institutional review board regulations, informed consent regulations, and the provisions of the Declaration of Helsinki. All parts of the study were approved by the local ethics committee of Friedrich-Alexander-Universität Erlangen-Nürnberg (FAU application number 84_19B). After detailed information about the risks and benefits of the study, written informed consent was obtained from all participants and patients.

### Cells

SV40-transformed human corneal epithelial cells (HCE cells, obtained from Kaoru Araki-Sasaki, Tane Memorial Eye Hospital, Osaka, Japan) (29), were cultured as monolayer and used for further experiments. Also human telomerase-immortalized corneal epithelial (hTCEpi) cells were cultured as monolayer as described before (30) and used for additional investigations concerning the corneal epithelium. Human Meibomian gland epithelial cell line (HMGEC) was cultured under standard conditions (37°C, 21% O2, 5% CO2) with and without 10% fetal calf serum to initiate differentiation as described before (31).

### Tissues

Lacrimal glands, upper and lower eyelids, conjunctivas, and corneas were obtained from human cadavers donated by written testamentary disposition to the Department of Functional and Clinical Anatomy of Friedrich Alexander University Erlangen-Nürnberg (FAU), Germany. All tissues were dissected from the cadavers within 4 to 12 h of death. Donors were free of recent trauma, eye and nasal infections, and diseases involving or affecting lacrimal apparatus or ocular surface function. After dissection, tissues from the eye of each cadaver were prepared for paraffin-embedding and were fixed in 4 % paraformaldehyde. Tissues for molecular biological investigations were immediately frozen at −80°C.

### RNA Preparation and Complementary DNA (cDNA) Synthesis

For conventional reverse transcriptase-polymerase chain reaction (RT-PCR), tissue samples of four lacrimal glands, four corneas, three conjunctivas, three nasolacrimal ducts and two eye lids (including Meibomian glands) were crushed in an agate mortar under liquid nitrogen, then homogenized in 5 ml RNA pure solution (peqgold; peqLab Biotechnologie, Erlangen, Germany) with a homogenizer (Polytron, Paterson, NJ, USA). Insoluble material was removed by centrifugation (12,000 g, 5 min, 4°C). Total RNA was isolated using RNeasy-Kit (Qiagen, Hilden, Germany). In addition, total RNA was extracted from cultivated HCE, hTCEpi and HMGEC cell lines by PeqGold reagent (PeQLab, Erlangen, Germany) according to manufacturer's protocol. Crude RNA was purified with isopropanol and repeated ethanol precipitation, and contaminated DNA was removed by digestion with RNase-free DNase I (30 min, 37°C; ThermoFisher Scientific, Waltham, MA, USA). The DNase was heat-denatured for 10 min at 65°C. Sample cDNA was generated from total RNA with the RevertAidTM Reverse Transkriptase-Kit of ThermoFisher Scientific (Waltham, MA, USA) according to the manufacturer's protocol. Two micrograms total RNA and 10 pmol Oligo (dT)18 primer (Fermentas) was used for each reaction. The cDNA was stored at −20°C until use.

### Reverse Transcriptase-Polymerase Chain Reaction (RT-PCR)

For RT-PCR we used ThermoFisher Scientific Kit (Waltham, MA, USA) according to manufacturer's protocol. At first, the integrity and stability of each transcribed cDNA was verified with PCR for human β-Actin (sense: GAT CCT CAC CGA GCG CGG CTA CA, antisense: GCG GAT GTC CAC GTC ACA CTT CA, annealing temperature 60°C, product 298 bp) before further analyses. For gene-specific PCR we used: 1 μL cDNA, 13.7 μl H_2_O, 1 μl 50 mM MgCl_2_, 0.5 μl dNTP, 2 μl 10 × PCR buffer, 0.3 μl (5 U) Taq DNA polymerase (Invitrogen, Karlsruhe, Germany), and 0.5 μl (100 pmol) of each of the following primers for conventional RT-PCR: Homo sapiens prolactin receptor (PRLR): NM_000949.6 (sense: AAG AGT GAA CAA GTG CAC CGA, antisense: AAG AGT GAA CAA GTG CAC CGA, annealing temperature 62°C, product 570 bp), homo sapiens prolactin inducible protein (PIP): NM_002652.2 (sense: GCT CAG GAC AAC ACT CGG AA, antisense: AAT CAC CTG GGT GTG GCA AA; annealing temperature 62°C, product 395 bp) and actin.

### Immunohistochemistry

Paraformaldehyde-fixed human cadaver tissue was embedded in paraffin, sectioned and dewaxed by descending alcohol series to xylol as described before (32). Immunohistochemistry was performed with polyclonal rabbit anti-prolactin receptor (1:50, abcam, ab170935, Cambridge, UK), mouse anti-prolactin (1:50, Novus Biologicals, NBP2-02142, Littleton, CO, USA), and goat anti-prolactin inducible protein (1:50, Invitrogen, PA518507, Carlsbad, CA, USA). Visualization was achieved with horseradish peroxidase-labeled streptavidin-biotin complex (StreptABComplex/HRP; Dako, Santa Clara, CA, USA) and 3-amino-9-ethylcarbazole (AEC; Dako, Santa Clara, CA, USA). Sections were counterstained with hemalum and mounted in Entellan (Dako). Sections were treated with the following standard treatments: 3% hydrogen peroxide, citrate buffer (pH 6) boiling, and Tris-buffered saline with Tween 20, the sections were incubated overnight at 4°C with primary antibodies and with secondary antibodies at room temperature for 2 h. Sections of pituitary gland, mammary gland and testis were used as positive controls. Negative control sections, incubated with non-immune IgG instead of primary antibody, were used in each case. All slides were examined with a Keyence BZ 9000 microscope.

### Tear Fluid Samples

Collection and analysis of human tear fluid was approved by the local ethics committee of FAU Erlangen-Nürnberg, Germany (FAU application number 84_19B). The study was also conducted in accordance with the tenets of the Declaration of Helsinki, compliance with good clinical practice and with informed consent. All subjects completed an institutional review board-approved questionnaire and underwent a general ophthalmological examination in accordance with the valid BVA (Berufsverband der Augenärzte Deutschlands [Professional Association of German Ophthalmologists]) and DOG (Deutsche Ophthalmologische Gesellschaft [German Ophthalmologic Society]) guidelines for Germany (https://www.dog.org/wp-content/uploads/2009/09/leit11.pdf). Healthy donors had no DED symptoms, no use of artificial tears, lubricants or re-wetting drops, no autoimmune disorders, and no other eye diseases, except for cataract or refractive errors. Subjects were considered to suffer from DED if they had a history of moderate DED with a documented diagnosis in their medical history by an ophthalmologist ≥6 months prior to study visit. Patients with DED were divided into three groups by fluorescein tear film break-up time (TBUT) and Schirmer type 1 (without anesthesia) method: (1) patients with aqueous deficient dry eye (ADDE) with Schirmer < 5 mm after 5 min, (2) patients with evaporative dry eye (EDE) with BUT < 5 s and (3) patients with the mixed dry eye (MDE) of evaporative dry eye (BUT < 5 s) plus aqueous deficient dry eye (Schirmer <5 mm after 5 min). Tear film break-up time (TBUT) was measured with 5 μl of non-preserved 2% sodium fluorescein as described before. Fluorescein solution was instilled onto the bulbar conjunctiva using a micropipette. Donors were instructed to blink normally without squeezing several times to distribute the fluorescein and then refrain from blinking until told otherwise. TBUT was measured by slit lamp magnification at 10-fold and a Wratten 12 yellow filter was used to enhance the observation of the tear film over the entire cornea. A stopwatch was used to record the time between the last complete blink and the first indication of tear film break-up. Thereafter, the patient was instructed to blink normally again. Three TBUT measurements were taken and the average was calculated. All patients were consecutively recruited in an established ophthalmology practice with specialized dry eye consultation hours in Nürnberg, Germany. Tear fluid samples were taken from 153 subjects (Table 1, 38 healthy donors, 115 DED patients) using Clement Clarke Schirmer tear test strips (Clement Clarke International Ltd, UK) without anesthetic (Schirmer I). Characteristics of patient study group are given in Table 1. The Schirmer tear test strips were immediately stored in a sterile reaction tube at −20°C until further processing. The tear fluid samples were extracted from the Schirmer strips with 60 μl 1x PBS via centrifugation by the adapted protocol as described before (33).

**Table 1:** Patients' characteristics and PIP tear concentration.

**Characteristics**	**Total**	**Healthy**	**DED**	**ADDE**	**EDE**	**MDE**
Number of subjects (n)	153	38	115	19	48	48
Sex						
Male: *n* (%)	53	20	33	1	21	11
Female: *n* (%)	100	18	82	18	27	37
Age: years						
Mean ± SD	49.1 ± 1.3	41.4 ± 2.8	51.5 ± 1.5	50.1 ± 3.8	48.1 ± 2.1	55.6 ± 2.2
PIP concentration / total protein (pg/mg)	515.5 ±	157.7 ±	633.8 ±	286.9 ±	470 ±	934.8 ±
Mean ± SEM	105.6	13.5	138.8	110.7	124.9	301.5
Minimum	17.2	46.7	17.2	38.0	17.2	20.6
Median	172.7	143.2	211.3	98.2	186.0	321.4
Maximum	11,292	383.6	11,292	1,952	4,522	11,292

*PIP concentration analyzed by PIP ELISA. n, number; yrs, years; SD, standard deviation; SEM, standard error of the mean*.

### ELISA

PRL and PIP concentration in the collected tear film samples were determined using the commercially available human PRL (KA0217, Anova, Taipei City, Taiwan) and PIP ELISA kit (EH2124, FineTest, Wuhan, China) according to the manufacturer's instructions. The PIP ELISA used has an average recovery range of 94% and precision in intra-assay < 8% and inter-assay < 10%. Cross-reactivity or interference between PIP and analogs is not observed. For ELISA 50 μL of each undiluted tear sample were used. The minimum detectable concentration by these assays is estimated to be 2 ng/ml for PRL and 18.8 pg/ml for PIP. The O.D. absorbance was read at 450 nm in a microplate reader (Clariostar, BMG Labtech, Ortenberg, Germany) and the concentration of PIP was calculated normalized to total protein amount of each tear sample.

### Statistical Analysis

All bar charts represent results and were plotted as mean ± standard error of mean (SEM). Gaussian distribution was calculated by the Kolmogorov–Smirnov test. After evaluating values for normal distribution, we performed unpaired students *t*-test or Mann Whitney test if we compared two groups as well as 1-way ANOVA statistics for more than two groups. For interpretation of the results, we used either Bonferroni or Dunn *post hoc* tests. Correlation statistics were calculated with Pearson or Spearman correlation coefficient. Charts were generated and statistical analyzed with GraphPad Prism (version 5). *P* < 0.05 were considered statistically significant.

## Results

### Gene Expression of Prolactin Receptor (PRLR) and Prolactin Inducible Protein (PIP) in Tissues of the Lacrimal Apparatus and Ocular Surface

**Figure 1:**
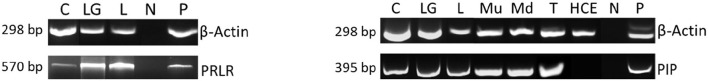
RT-PCR analysis of PRLR and PIP gene expression in human ocular tissues and cell lines. The expression of β-actin gene served as internal control for assessing integrity and stability of the transcribed cDNA. Cornea (C), lacrimal gland (LG), eye lid (L), undifferentiated human Meibomian gland epithelial cell line (Mu), differentiated human Meibomian gland epithelial cell line (Md), human corneal epithelium cell line hCTEpi (T), human corneal epithelium cell line HCE (HCE), negative control (N) positive control (P) for PRLR is submandibular gland and for PIP is testis. Pictures represent three independent experiments.

To analyze the gene expression of PRLR and PIP in the lacrimal apparatus and ocular surface RT-PCR was performed with human tissues of the lacrimal gland, cornea and eyelid obtained from cadavers and also with immortalized corneal epithelial cell lines (HCE and hTCEpi) and the human Meibomian gland epithelial cell line HMGEC. Our RT-PCR analyses revealed gene expression of PRLR in the lacrimal gland, cornea, and eyelid (Figure 1). There was no expression of PRLR transcript in human cell lines of corneal epithelium (HCE and hTCEpi) and human meibomian gland epithelial cells (HMGECs) (data not shown). A PIP transcript was present in the lacrimal gland, cornea, and eyelid from human cadavers as well as in hTCEpi and HMGEC cultivated in serum-free and serum-containing medium. No expression of PIP was detectable in the human corneal epithelium cell line HCE (Figure 1).

### Localization of PRL, PRLR and PIP in Tissues of the Ocular Surface and Lacrimal Apparatus

To verify gene expression and localize the expression of PRL, PRLR and PIP at the ocular surface and lacrimal apparatus, immunohistochemical (IHC) analyses of formalin-fixed paraffin-embedded tissue sections from 10 human cadavers were performed for each tissue. The representative distribution in the analyzed tissues was as follows.

**Figure 2:**
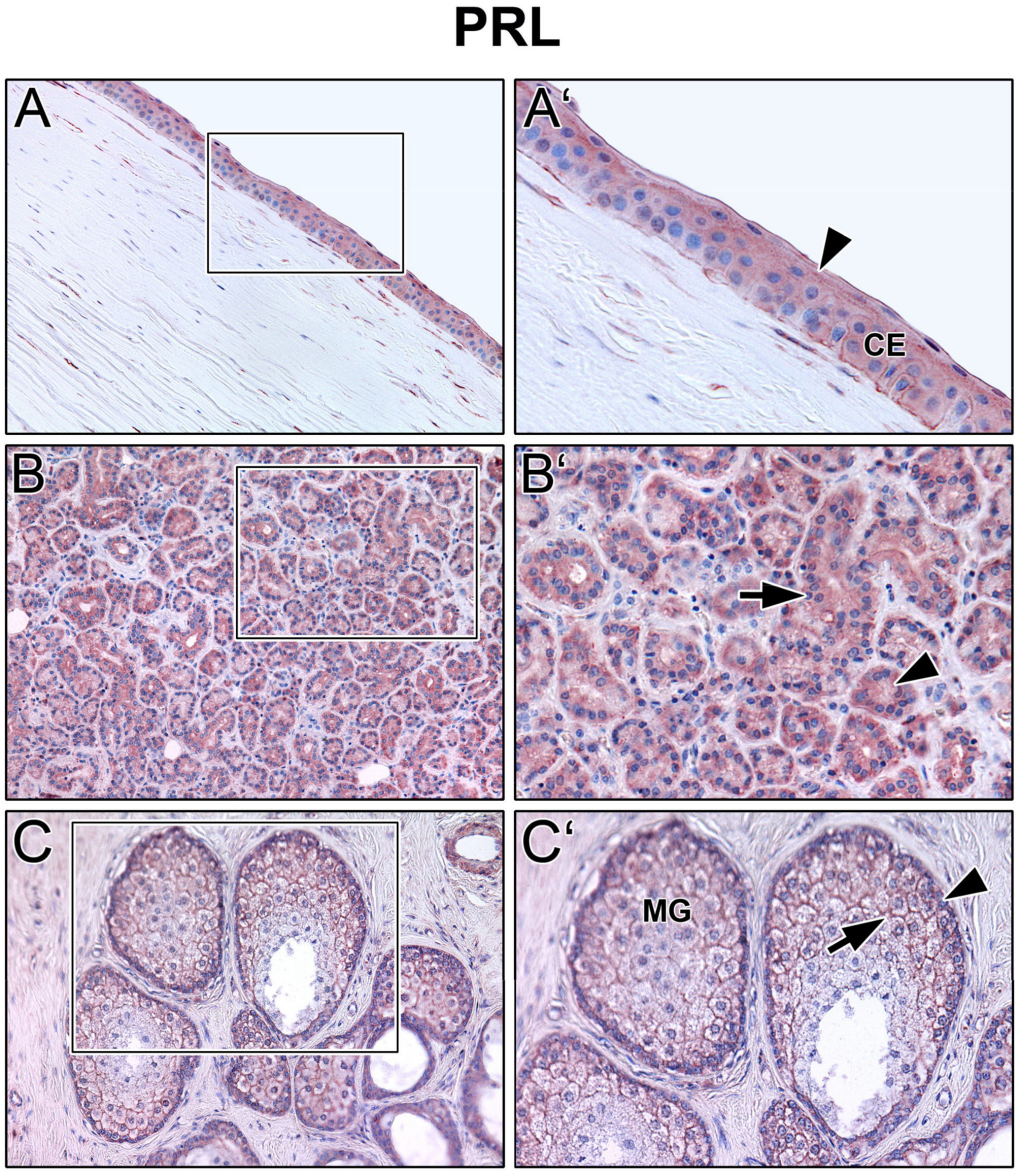
Immunohistochemical analyses of prolactin (PRL) in the human cornea **(A)** with corneal epithelium (CE), lacrimal gland **(B)** and eyelid **(C)** with Meibomian gland (MG). The antibody reaction is visible by the intracellular red reaction product. Pictures represent meaningful immunohistochemical analyses of sections obtained from 10 different cadavers for each tissue (*n* = 10). **(A,B)** and **(C)** Inlays show higher magnification. Nuclei are counterstained with hemalum (blue). Arrows and Arrowheads accentuate the reactivity localization.

**Figure 3:**
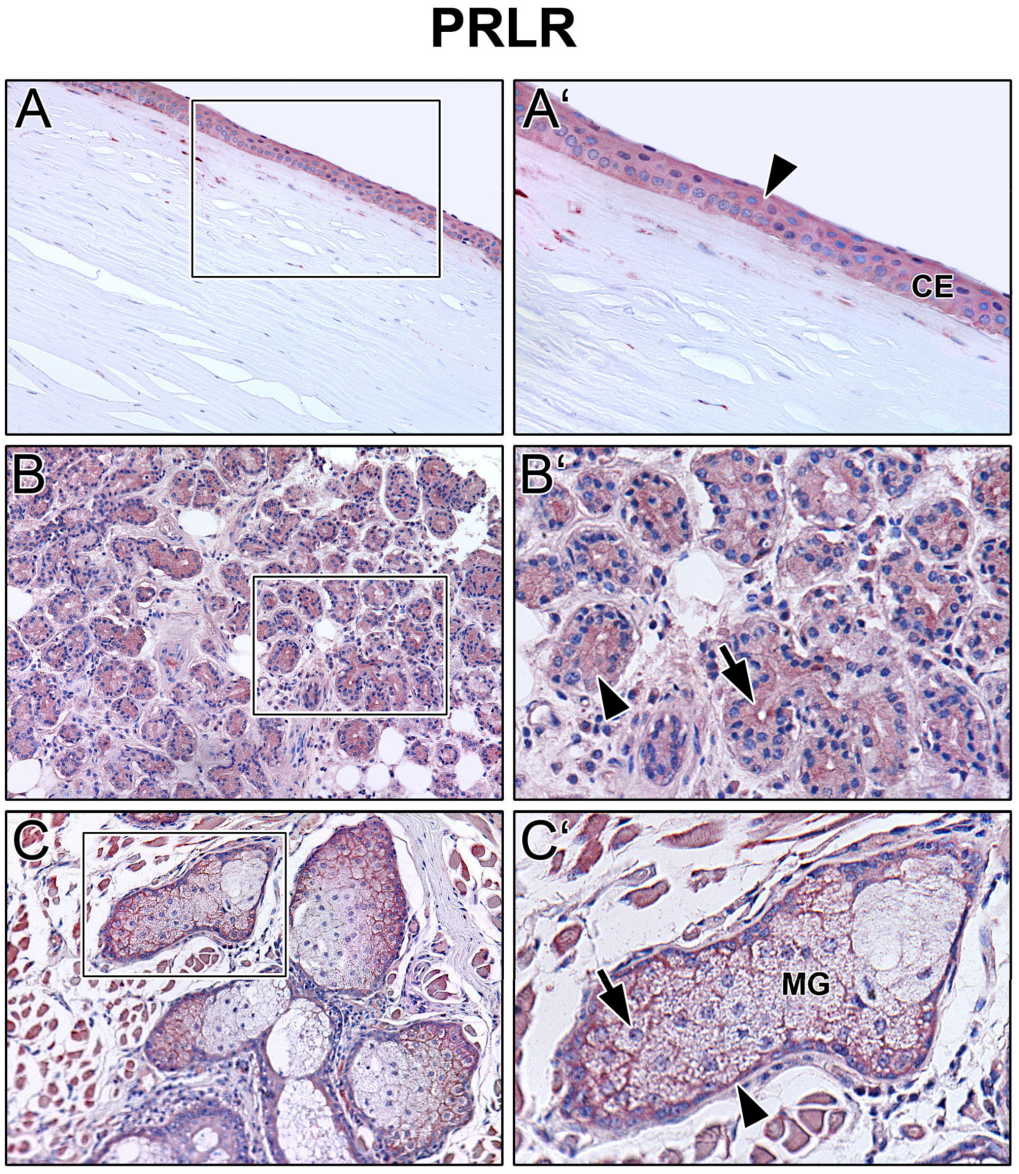
Immunohistochemical analyses of prolactin receptor (PRLR) in the human cornea **(A)** with corneal epithelium (CE), lacrimal gland **(B)** and eyelid **(C)** with Meibomian gland (MG). The antibody reaction is visible by the intracellular red reaction product. Pictures represent meaningful immunohistochemical analyses of sections obtained from 10 different cadavers for each tissue (*n* = 10). **(A,B)** and **(C)** Inlays show higher magnification. Nuclei are counterstained with hemalum (blue). Arrows and Arrowheads accentuate the reactivity localization.

**Figure 4:**
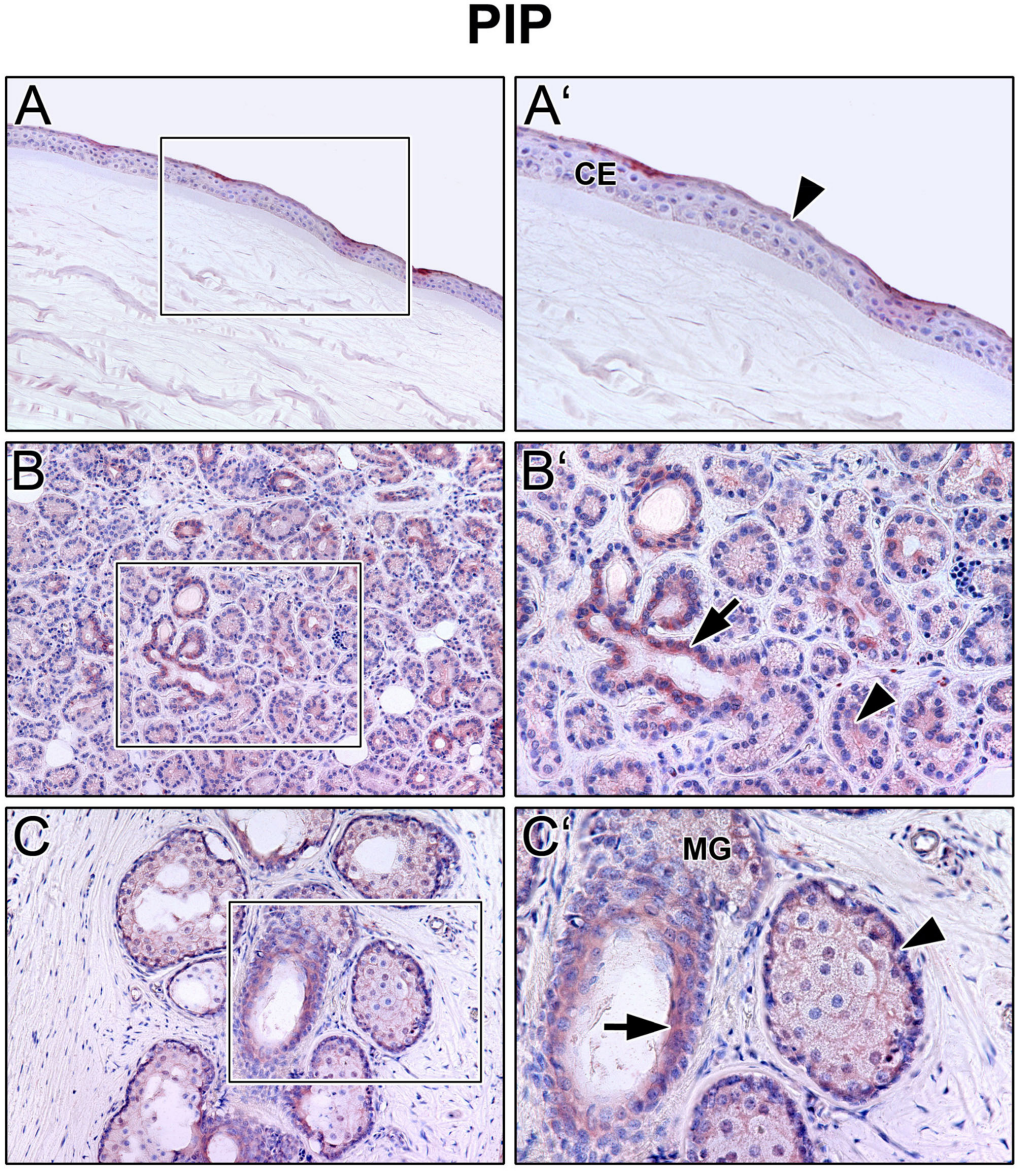
Immunohistochemical analyses of prolactin inducible protein (PIP) in the human cornea **(A)** with corneal epithelium (CE), lacrimal gland **(B)** and eyelid **(C)** with Meibomian gland (MG). The antibody reaction is visible by the intracellular red reaction product. Pictures represent meaningful immunohistochemical analyses of sections obtained from 10 different cadavers for each tissue (*n* = 10). **(A,B)** and **(C)** Inlays show higher magnification. Nuclei are counterstained with hemalum (blue). Arrows and Arrowheads accentuate the reactivity localization.

Immunoreactivity was visible with PRL antibody in all cell layers of the corneal epithelium as well as in fibroblasts and free cells of the corneal stroma. The tuboalveolar acinus cells and peripheral immune cells in the lacrimal gland showed a strong intracytoplasmic immunoreactivity. PRL was also detectable in the upper and lower eyelid, especially in the acinus cells of Meibomian glands (Figure 2). Moreover, prolactin receptor (PRLR) revealed comparable localization patterns in the conducted immunohistochemistry analyses. PRLR was visible in corneal epithelial as well as stromal cells of human cornea. In addition, tuboalveolar acinus cells of the lacrimal gland showed strong immunoreactivity. The same applied for the eyelids, especially the cell membranes of basal and mature meibocytes revealed a reactivity, but not hypermature and apoptotic meibocytes. Additionally, the surrounding striated skeletal muscle cells of the Meibomian glands, Riolan part of the orbicularis oculi muscle, indicated strong reactivity (Figure 3). The presence of PRLR in skeletal muscles but also in smooth muscles is already known (12, 34–36). Immunoreactivity with PIP antibody was clearly visible in apical corneal epithelial cells whereas intermedial and basal corneal epithelial cells only showed a weak reactivity. No immunoreactivity could be detected in cells of the corneal stroma. In addition, tuboalveolar acinus and immune cells of the lacrimal gland showed strong intracytoplasmic immunoreactivity. PIP was present in the cell membrane and nuclei of non-apoptotic meibocytes of upper and lower eye lids as well (Figure 4).

### Increased Concentration of Prolactin Inducible Protein (PIP) in Reflex Tears From DED Patients

**Figure 5:**
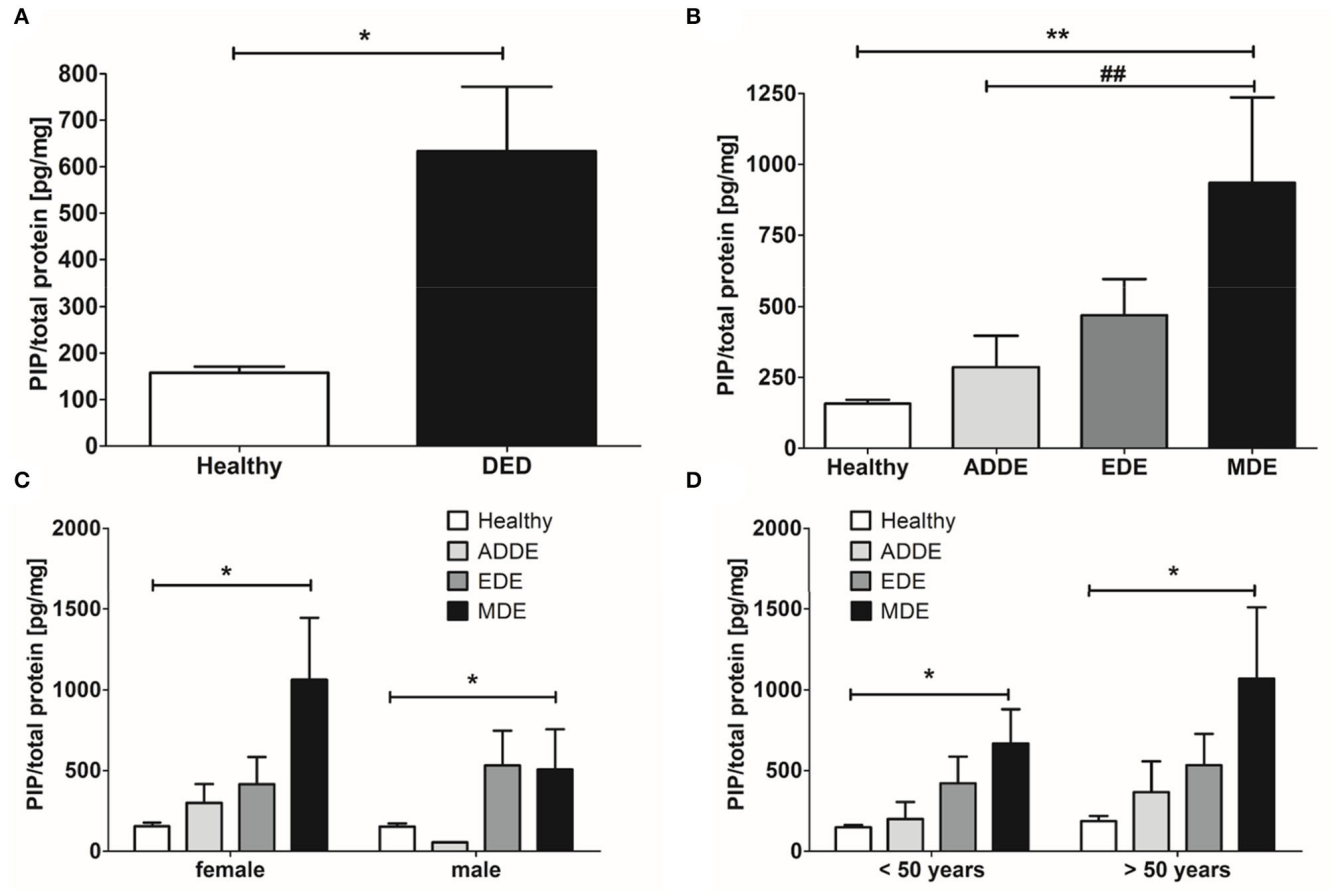
ELISA analyses of PIP in human tear samples. **(A)** Significant increase of the PIP concentration in tear samples from patients with dry eye disease (DED, *n* = 115) compared to healthy donors (*n* = 38). **(B)** PIP concentration in DED subgroups. There are increased PIP levels in tear samples of patients with aqueous deficient dry eye (ADDE, *n* = 19), evaporative dry eye (EDE, *n* = 48) and mixed dry eye (MDE, *n* = 48) compared to healthy subjects. Significantly increased PIP levels can only be seen in MDE compared to healthy subjects and in MDE compared to ADDE. **(C)** PIP concentration in analyzed groups divided by sex (women, *n* = 100 and men, *n* = 53). Significantly increased PIP levels can only be seen in MDE compared to healthy subjects. **(D)** PIP concentration in analyzed groups divided by age. There are significantly increased PIP levels in MDE compared to healthy subjects. One way anova and Dunn multiple comparison test, *p < 0.05, **/^##^*p* < 0.01.

We collected reflex tears from healthy donors as well as patients with DED and measured PRL and PIP concentration in both groups. For this purpose, we first analyzed 50 μl tear samples with a PRL ELISA and then the same sample with a PIP ELISA. In none of the analyzed reflex tear samples PRL could be detected above the detection limit of the ELISA used (data not shown). Furthermore, we analyzed PIP concentration in tear samples of DED patients and healthy donors by ELISA (Table 1). Our results showed a significant 400% increase in PIP concentration in tears from dry eye patients (DED, 633.8 ± 138.8 pg/mg total protein, p = 0.043) compared to tears from healthy donors (157.7 ± 13.5 pg/mg, Figure 5A). In three common DED subgroups PIP concentration was measured: Aqueous deficient dry eye (ADDE) with 286.9 ± 110.7 pg/mg, evaporative dry eye (EDE) with 470 ± 124.9 pg/mg and mixed dry eye (MDE) with 934.8 ± 301.5 pg/mg. The MDE subgroup showed a significant increase in PIP concentration compared to tears from healthy donors or ADDE patients (both *p* < 0.01). There was no significant difference of PIP concentration in tears from EDE and ADDE patients (Figure 5B). Additionally, there was a significant PIP increase in the female MDE subgroup (1,062 ± 383.2 pg/mg, *p* = 0.0036) as well as in the male MDE subgroup (506.9 ± 249.4 pg/mg, *p* = 0.049) compared to the sex controls. ADDE and EDE showed no significant difference in PIP concentration as a function of sex. PIP concentration in MDE was significantly different between genders but not statistically significant (*p* = 0.3641). No significant differences were found in the other DED subgroups and in healthy subjects (Figure 5C). The total protein amount in all tear samples from DED patients (1.32 ± 0.10 μg/μl) showed a significant decrease of about 20% compared to healthy donors (1.70 ± 0.12 μg/μl, *p* = 0.0009). Total protein levels specifically were not significantly decreased in ADDE (1.37 ± 0.27 μg/μl) and EDE (1.46 ± 0.15 μg/μl), while they were significantly lower in the MDE subgroup (1.16 ± 0.15 μg/μl; *p* < 0.001) (Supplementary Figure S1).

Furthermore, the extant of the influence that the age of different doners has on the PIP concentration was investigated. Therefore, data from individuals under and over the age of 50 was analyzed. The results showed a similar trend in both age groups. All DED subgroups showed increased PIP concentration compared with healthy donors (Figure 5D). In donors younger than 50 years with ADDE, the concentration was increased 1.3-fold to 199.3 ± 105.9 pg/mg and 2.8-fold to 420.6 ± 165.7 pg/mg in donors with EDE compared with healthy donors at 148.8 ± 14.6 pg/mg. In donors older than 50 years, PIP levels increased 2.0-fold to 365.8 ± 190.2 pg/mg in ADDE patients and 2.9-fold to 533.5 ± 193.8 pg/mg in the EDE subgroup compared with the age-matched healthy group at 186.7 ± 31.6 pg/mg. However, PIP concentration only increased significantly in the MDE subgroup: by 4.5-fold to 666.7 ± 212.5 pg/mg (*p* < 0.05) in subjects younger than 50 years and by 5.7-fold to 1,069 ± 440.6 pg/mg (p < 0.05) in subjects older than 50 years compared with the age-matched healthy group.

**Figure 6:**
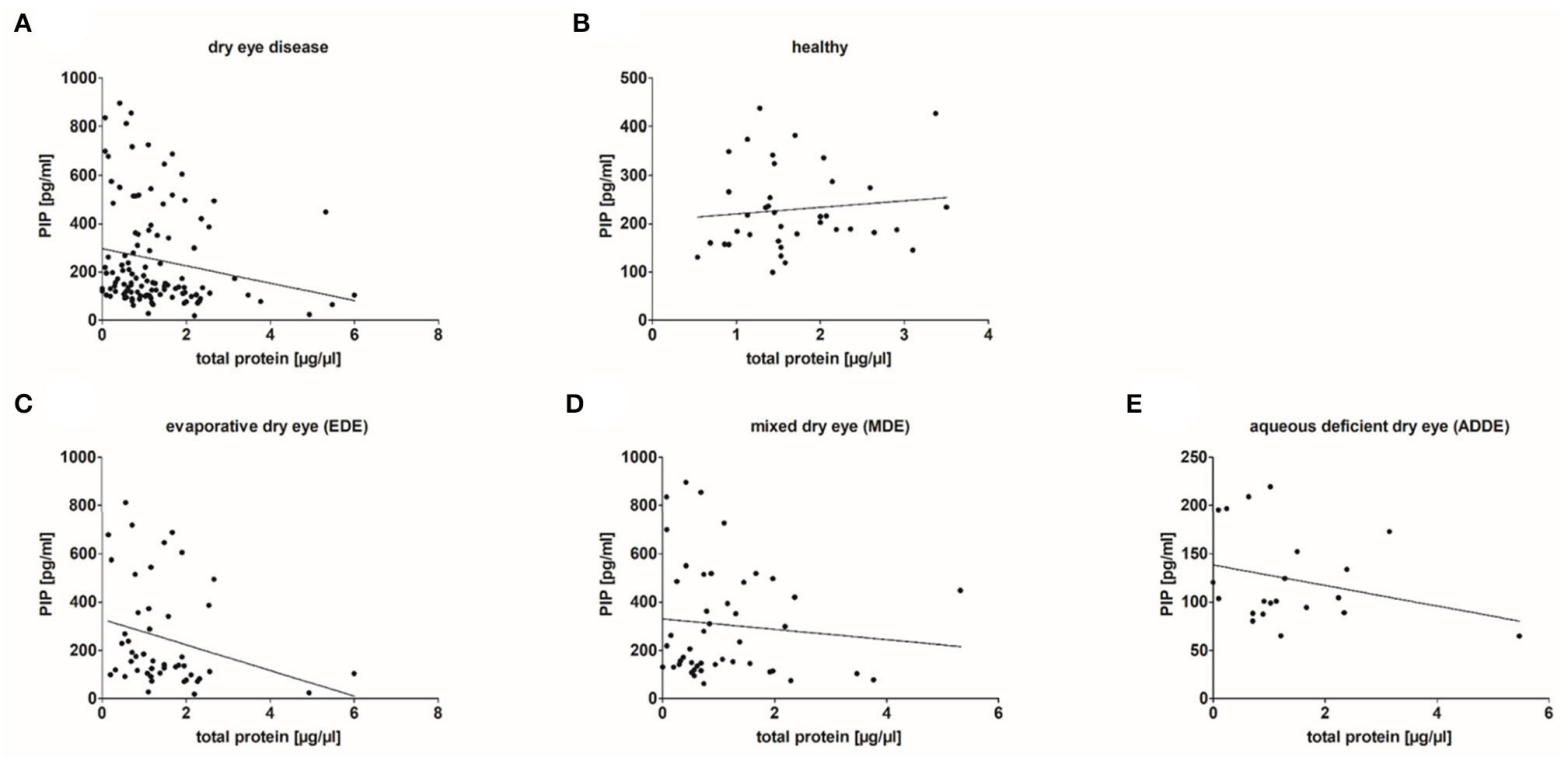
Correlation of PIP concentration and total protein amount in analyzed tear samples. **(A)** Results show a significant weak negative correlation between PIP and the total protein amount in tear samples from dry eye patients (*n* = 115; r = −0.261, *p* = 0.005). **(B)** No correlation in tears obtained from healthy donors (*n* = 38). **(C)** A significant negative correlation in tears from evaporative dry eye patients (*n* = 48, r = −0.351, *p* = 0.015). **(D,E)** No significant correlation in tears from mixed (*n* = 48) and aqueous dry eye patients (*n* = 19). Correlation statistic is calculated with Spearman correlation coefficient.

In addition, PIP concentration in tear samples was correlated with total protein amount in the different groups (Figure 6). The results showed a weakly significant negative correlation between PIP concentration and total protein amount in tear samples from dry eye patients (*n* = 115; r = −0.261, *p* = 0.005). Tear samples from healthy donors showed no correlation between PIP and total protein (*n* = 38, r = 0.091, *p* > 0.05). A closer look at the underlying subgroups revealed a significant negative correlation only in EDE patients (*n* = 48, r = −0.351, *p* = 0.015) (Figure 6C) whereas MDE (r = −0.101; *p* > 0.05) and ADDE (r = −0.201; *p* > 0.05) did not show significant differences in correlation between PIP and total protein in tears by themselves (Figures 6D,E).

## Discussion

The present results show that prolactin (PRL), prolactin receptor (PRLR), and prolactin inducible protein (PIP) are expressed in the human lacrimal apparatus and on the ocular surface (Figures 1–4). In addition, PIP is a soluble component of the tear film and may contribute to tear quality in DED (Figure 5). Decreased production of the aqueous component of the tear film and evaporation of the tear film are common causes of DED (4). Besides age, sex is a relevant risk factor for DED, which is more common in women than in men. It is well known and clinically relevant that female gender is a significant risk factor for the development of DED (37, 38). According to the TFOS DEWS II Sex, Gender, and Hormones Report, gender differences in DED prevalence are primarily due to the effects of sex hormones, particularly androgens and estrogen (2, 4, 6, 39, 40). Moreover, other hormones like hypothalamic-pituitary hormones, e.g., PRL, and thyroid hormones have an impact on the pathogenesis of DED (6). Our immunohistochemical results show that PRL and PRLR are expressed in the tissues of the lacrimal apparatus, including the corneal epithelium, lacrimal gland, and Meibomian glands in the eyelid at the protein level in humans (Figures 2, 3). These results confirm the findings of previous studies in laboratory animals, like Sprague-Dawley rats and New Zealand White rabbits, in which the expression of PRL and PRLR was detected in the acinar cells of the lacrimal gland (14, 15, 41). Our ELISA analyses of reflex tear samples from DED patients and healthy donors using a commercial diagnostic ELISA kit for PRL showed the absence of PRL in tears or at least a PRL concentration below the detection limit of the ELISA kit (data not shown). Pituitary PRL is produced mainly by endocrine neurons in the hypothalamus, but also by various other cells and tissues, including the lacrimal system (14, 15, 41). It is a multifunctional hormone that is regularly present in tears of laboratory animals such as mouse, rat and rabbit (12–15). In these animals, PRL plays a minor role in the sexual dimorphism of the lacrimal gland of mice and rats, including morphology, secretion profile, and tear volume (14, 42, 43). In hypophysectomized female rats, PRL treatment partially restored protein levels in the gland, particularly Na^+^/K^+^-ATPase activity, alkaline phosphatase activity, and the number of cholinergic receptors (44). Our immunohistochemical results showed no sex-specific differences in the morphology and expression patterns of PRL, PRLP, and PIP in all tissue sections examined, which were derived from female and male cadavers. The functional role of PRL, sex differences in lacrimal glands and effects on tear film stability and dynamics are not yet known in detail and require further investigation.

Studies about PLR in human tears are limited (16, 17). Our own PRL ELISA results showed the absence of PRL in human tears (s.a.). In contrast, PRL blood serum concentration has been shown to correlate negatively with tear quality in women on hormone replacement therapy (18). In another study, women, but not men, with seborrheic MGD revealed significantly higher PRL levels in blood serum compared to controls (45). Both studies did not include PRL concentration or other hormonal factors in the donor tears. In accordance with our PRL immunohistochemistry results (Figure 2) showing PRL expression in the lacrimal apparatus, as well as published results detecting PRL in lacrimal glands and tears from laboratory animals, it is conceivable that PRL is present in human tears, but at a much lower concentration, at least below the detection limit of the PRL ELISA used (~2 ng/ml). It is mentioned that PRL levels differ during the day with low levels at nighttime and are affected by acute stress, through food intake, various diseases and medication (46, 47). Furthermore, it is well known that reflex and basal tears show different composition beside different function at the ocular surface. We can therefore not rule out that the basal tears contain PRL in higher or measurable concentrations. Further investigations are necessary to answer this question.

Binding of PRL to the prolactin receptor (PRLR) induces an increased expression of prolactin inducible protein (PIP) (20). PIP on the other hand, leads (among others) to an increased placement of the water channel aquaporin 5 (AQP5) into the apical cell membrane of mouse lacrimal glands (48). This selective water channel AQP5 is well characterized in the lacrimal gland and corneal epithelium (49, 50). A loss of AQP5 in the lacrimal gland and corneal epithelium could theoretically lead to a reduction of the aqueous component of the tear film with consecutive aqueous deficient dry eye (ADDE). It has already been shown in a mouse model that a lack of AQP5 in the lacrimal gland and salivary gland seem to influence the aqueous component of the tear film and contribute to the autoimmune form of DED Sjögren's syndrome (48) and immunohistochemical examination of the human lacrimal gland in severe forms of dry eye due to Stevens-Johnson syndrome have demonstrated loss of AQP5 (51). In another study, tear proteome analysis showed that PIP is still significantly downregulated in stably controlled Sjögren's syndrome DED patients (52). Additionally, Zhou et al. demonstrated in a small group of DED patients a decreased PIP concentration in tears by isobaric tag for relative and absolute quantitation (iTRAQ) technology (53). Further proteomic analyses revealed a decreased PIP amount in saliva of patients with primary Sjögren's syndrome, so that PIP can function as a potential biomarker for Sjögren's syndrome (28, 52, 53). Only an insufficient number of Sjörgen's syndrome patients were involved in the patient cohort of our PIP ELISA test (s.a.), so that we cannot make any statistically reliable statements about the PIP concentration. In fact, we wanted to investigate the PLR induced PRLR activated downstream signaling of PIP and AQP5 at the ocular surface. Nevertheless, our RT-PCR results showed no gene expression of PRLR in two well-established human corneal epithelium cell lines HCE and hCTEpi (Figure 1). Even after stimulating HCE and hCTEpi with PRL up to 72 h, there was no gene expression of PRLR (data not shown). As of now, we could not analyze the possible signaling pathway of PRLR, PIP, and AQP5 *in vitro*. This finding limits the outcome of the molecular interaction and regulation of PRL, PRLR, and PIP associated proteins like AQP5 in our study. Further studies with primary corneal epithelial cells or *in vivo* models are necessary here.

However, our ELISA analyses of PIP in human tear fluid samples show a significantly increased concentration in patients with DED up to fourfold. Specifically, the ADDE group shows a 1.8-fold increase, the EDE group a 3.0-fold increase, and the MDE subgroup shows the largest increase with a 5.9-fold increase compared to healthy donor tears (Figures 5A,B). This result is initially surprising in view of the recommended studies in ADDE patients and in the rabbit model with autoimmune Sjögrens syndrome, in which a lower concentration of PIP in tears has been demonstrated (52, 53). A lower concentration of PIP in tears and other body fluids has also been demonstrated in patients with keratoconus, an ectatic corneal disease, and highlights the role of PIP as a novel keratoconus biomarker (25–27). In patients with keratoconus, PIP expression in primary keratoconus cells as well as tears, serum, and saliva is markedly downregulated regardless of age, sex, and severity of keratoconus disease (25, 26). Our PIP results and these clinical studies show that sex hormones and sex hormones regulated factors like PIP influence the ocular surface in different manners and imbalances might cause different diseases of the ocular surface. For our ELISA experiments, we used reflex tears by collecting tears with Schirmer strips without anesthesia (Schirmer 1) in DED and control group. It is mentioned that reflex and basic tears are different in composition and function. This aspect should be analyzed in a further follow up study.

In our patient cohort, the subdivision of the DED patients according to sex and age shows a distribution typical for DED. The proportion of women with DED is higher and the number of DED patients increases with age. Our data also show that the increase in PIP concentration is very similar in men and women, even among different subtypes (Figure 5C). In the male cohort, the ADDE group is very small and does not allow us to draw a significant conclusion. Surprisingly, the PIP concentration is similarly distributed in both cohorts, regardless of the selected age classification. Age seems to have little influence on PIP concentration, but the underlying disease form of DED does (Figure 5D). Thus, our results indirectly show the distribution of PIP in the DED subgroups and the relationship between them. In addition to the non-DED samples, the ADDE groups have the lowest average PIP concentration compared with EDE and MDE. ADDE is a typical age-related DED due to damage to the lacrimal gland and reduced ocular surface evaporation (54). In ADDE, patients do not seem to produce more PIP to compensate for disturbances in aqueous secretion of the lacrimal gland. Furthermore, our results show that PIP concentration is negatively correlated with total protein content in DED, especially in EDE patients, but not in healthy subjects (Figure 6). In this context, it was noticed that there is a lower total protein concentration in tears from DED, especially in MDE patients (Supplementary Figure S1). This is consistent with previous studies. Versura et al. show that tears from patients with early DED have a significant reduction in tear protein, associated with a decrease in proteins with antibacterial and protective functions (55). This repeatedly demonstrates that not only the pure tear quantity but also, as in PIP, the concentration and certainly the composition of the tear proteins changes significantly in DED.

However, it is well known that in DED patients, a significantly higher proportion of patients show signs of EDE than ADDE (56). Meibomian gland dysfunction (MGD) is the common reason for EDE, which describes a chronic abnormality of the Meibomian glands, characterized by terminal duct obstruction and/or qualitative/quantitative changes in lipid secretion (37, 57). Interestingly, MGD leads to local inflammation of the ocular surface, and inflammatory conditions such as autoimmune diseases can trigger MGD (3, 58, 59). This leads to another important functional aspect of PRL and PIP. Both factors show a high relevance in immunological and autoimmune processes (60). The endocrine/paracrine PRL has been shown to stimulate immune cells by binding to PRLR (60). Increased PRL levels could depend on the enhancement of coordinated bi-directional communications between PRL and the immune system. Even though PRL is described as immunostimulatory or -protective, there is evidence that PRL is immunosuppressive at higher concentrations and that inappropriate prolongation of PRL synthesis could lead to autoimmune diseases (10, 13, 61). PIP is also important for immunomodulation, cell-mediate adoptive immunity and inflammation processes (23, 62, 63). This knowledge, our findings of an increased concentration of PIP in tears of EDE and MDE patients (Figure 5A), and the presence of PRLR and PIP in the Meibomian gland (Figures 3, 4) support the hypothesis that higher levels of PRL and PIP in tears also contribute to EDE and MDE via an unknown mechanism. This provides an incentive to further analyze the autoimmune and inflammatory character of the PRL and PIP signaling cascade in future DED research. Furthermore, it would be interesting if PIP or PRL are present in meibum oil and if concentration correlate with Meibomian gland acini anatomy in MGD patients. This could be analyzed by further meibography studies. In accordance with the anatomy, tear fluid drains from the ocular surface through the nasolacrimal ducts into the inferior meatus of the nose (64). Another progressive inflammatory syndrome of unknown etiology and predominantly affecting post-menopausal females is primary acquired nasolacrimal duct obstruction (PANDO). Recently, we were able to show that PRL and PIP play a role in the etiopathogenesis of lacrimal drainage obstructions (62, 63, 65). There might be a connection of increased PIP concentration in the tear fluid due to DED and PANDO that should also be analyzed in future studies.

In summary, our results demonstrate the expression of PRL, PRLR and PIP in human cornea, lacrimal gland, and Meibomian gland. Additionally, we show that the PIP concentration is increased in reflex tears of DED patients with mixed DED. The different roles of PRL and PIP in diseases of the ocular surface, each form of DED and especially in autoimmune Sjögren's syndrome need to be analyzed in further studies. Furthermore, regarding possible immunomodulatory functions of PRL and PIP-induced signaling cascades, additional investigations are necessary to learn more about the impact of both factors in tear film composition and quality. Understanding the role of those factors could provide new targets for diagnostic or therapeutic use in the treatment of DED.

## Author's Note

The present work was performed in fulfillment of the requirements for obtaining the degree “Dr. med.” at the medical faculty of the Friedrich-Alexander-Universität Erlangen-Nürnberg (FAU).

## Data Availability Statement

The raw data supporting the conclusions of this article will be made available by the authors, without undue reservation.

## Ethics Statement

The studies involving human participants were reviewed and approved by Ethics Committee of Friedrich-Alexander-Universität Erlangen-Nürnberg (FAU) (application number 84_19B). The patients/participants provided their written informed consent to participate in this study.

## Author Contributions

KJ performed most of the experimental work. FG performed ELISA experiments and mainly contributed to the conception and design of the study. KJ and FG performed the statistical analysis and wrote the first draft of the manuscript. CJ and JH-W were responsible for tear sample collection. All authors have contributed directly to the planning, execution, analysis of the work reported, manuscript revision, read, and approved the submitted version.

## Funding

KJ received support by a scholarship from the Association of German Ophthalmologists (DOG) in the year 2019 as well as Sicca Forschungsförderung of the Professional Association of German Ophthalmologists (BVA). FP was supported by the Deutsche Forschungsgemeinschaft grant PA738/15-1. FG was partly supported by Ernst-Muck Foundation FAU Erlangen-Nürnberg.

## Conflict of Interest

The authors declare that the research was conducted in the absence of any commercial or financial relationships that could be construed as a potential conflict of interest. The reviewer SS declared a past co-authorship with one of the author FP to the handling editor.

## Publisher's Note

All claims expressed in this article are solely those of the authors and do not necessarily represent those of their affiliated organizations, or those of the publisher, the editors and the reviewers. Any product that may be evaluated in this article, or claim that may be made by its manufacturer, is not guaranteed or endorsed by the publisher.

## Abbreviations

DED: dry eye disease
PLR: Prolactin
PRLR: prolactin receptor
PIP: prolactin inducible protein
HCE: human corneal epithelial cell line
HMGECs: human Meibomian gland epithelial cells.
