# Supplementary figures and images for: Prolactin Inducible Protein, but Not Prolactin, Is Present in Human Tears, Is Involved in Tear Film Quality, and Influences Evaporative Dry Eye Disease
Source: Front Med (Lausanne). 2022 Jun 30;9:892831. doi: 10.3389/fmed.2022.892831 (PMC9279896; doi:10.3389/fmed.2022.892831)

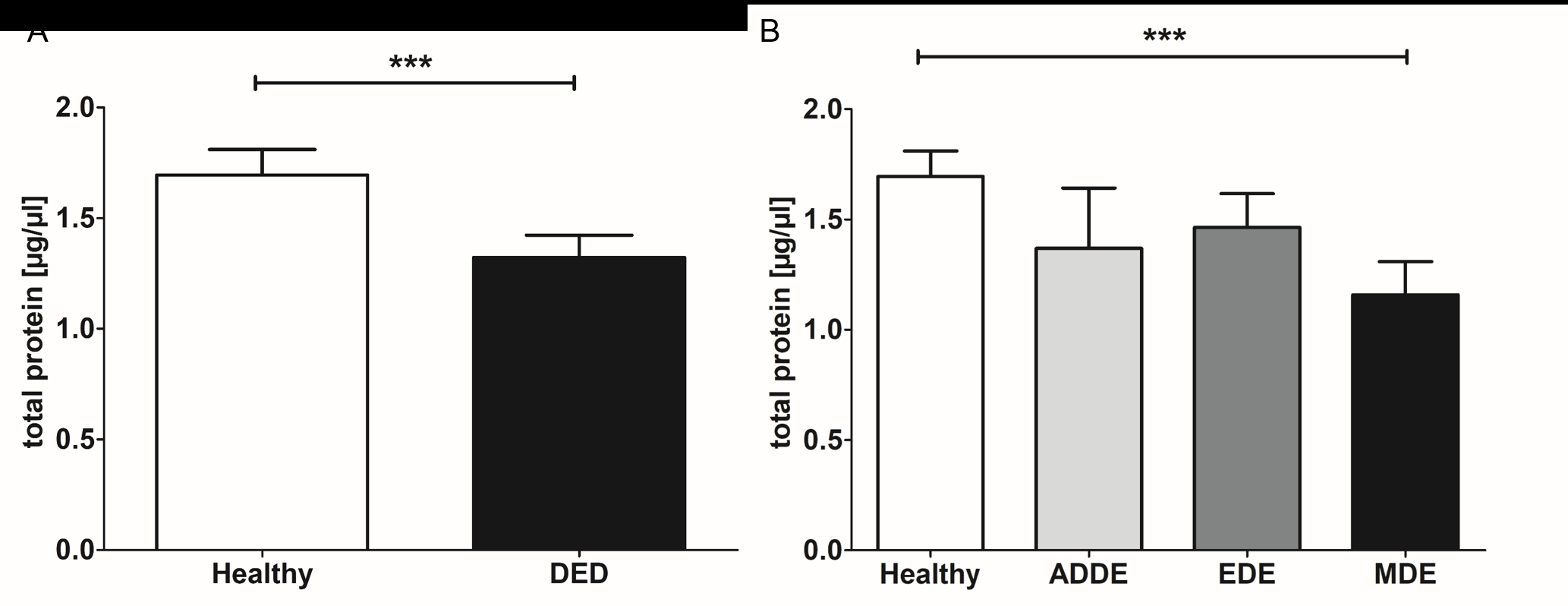

Supplement: Supplementary file 1 [file Image_1.tif]
